# Advanced Heterogeneous Feature Fusion Machine Learning Models and Algorithms for Improving Indoor Localization †

**DOI:** 10.3390/s19010125

**Published:** 2019-01-02

**Authors:** Lingwen Zhang, Ning Xiao, Wenkao Yang, Jun Li

**Affiliations:** 1 School of Electronics and Information Engineering, Beijing Jiaotong University, Beijing 100044, China; zhanglw@bjtu.edu.cn (L.Z.); wkyang@bjtu.edu.cn (W.Y.); 2 Tandon School of Engineering, New York University, New York, NY 11201, USA; jl7333@nyu.edu

**Keywords:** indoor localization, heterogeneous features fusion (HFF), machine learning, optimization

## Abstract

In the era of the Internet of Things and Artificial Intelligence, the Wi-Fi fingerprinting-based indoor positioning system (IPS) has been recognized as the most promising IPS for various applications. Fingerprinting-based algorithms critically rely on a fingerprint database built from machine learning methods. However, currently methods are based on single-feature Received Signal Strength (RSS), which is extremely unstable in performance in terms of precision and robustness. The reason for this is that single feature machines cannot capture the complete channel characteristics and are susceptible to interference. The objective of this paper is to exploit the Time of Arrival (TOA) feature and propose a heterogeneous features fusion model to enhance the precision and robustness of indoor positioning. Several challenges are addressed: (1) machine learning models based on heterogeneous features, (2) the optimization of algorithms for high precision and robustness, and (3) computational complexity. This paper provides several heterogeneous features fusion-based localization models. Their effectiveness and efficiency are thoroughly compared with state-of-the-art methods.

## 1. Introduction

With seamless integration of the physical world and the digital world through networks, the era of the Internet of Things (IoT) beckons. It offers a tremendous amount of opportunities for numerous novel applications that contribute to a significantly improved daily life [1]. With the surge in demand for location services, Location-Based Services (LBSs) become one of the key applications. For outdoor localization under the Line-Of-Sight (LOS) propagation conditions, the Global Positioning System (GPS) has matured and been successfully applied in various fields. For indoor localization under the None-Line-Of-Sight (NLOS) propagation conditions, extending the GPS to indoor environments is extremely difficult due to irregular signal fading and multi-path interference [2]. Therefore, indoor localization requires innovative solutions.

Typically, existing indoor positioning methods can be divided into three categories: the affinity method, the geometric-based method and the fingerprint method. With the affinity method, the location of the target node is approximated by the location of the service node when the mobile target accesses the service node at the known location. The affinity method has low computational complexity yet poor localization precision. Geometry-based positioning methods are either time based using the time-of-arrival (TOA) or time-difference-of-arrival (TDOA) methods [3], or by using the angle-of-arrival (AOA) methods [4] or Received Signal Strength (RSS)-based methods [5,6]. The accuracy of localization depends on the accuracy of the measurement [7,8]. Since the measurements are always disturbed by additive noise, multipath fading, shadowing and other interferences, localization is not reliable [9]. More reliable techniques for RSS-based localization are based on fingerprinting [10]. However, they need a preconfiguration phase where a collection of fingerprints is stored. The advantage of the fingerprinting technique is that it takes into account the stationary characteristics of the environment, such as multipath propagation and wall attenuation [11].

Most of the current fingerprint positioning technologies are based on single channel characteristics. However, for the sake of using only the single channel feature, once the channel feature is subject to greater interference, the precision in positioning will also decrease by a wide margin. Furthermore, artificial intelligence (AI) technology has been successfully applied in various fields, the indoor localization field being one such field. The motivation of this paper is to develop an enhanced fingerprint localization technology based on heterogeneous feature fusion combined with machine learning.

### 1.1. Related Works

The emerging techniques in the Fifth Generation (5G) communication system enable us to measure the angle-of-arrival (AOA), RSS and time-of-arrival (TOA) with various types of mobile devices [12,13]. RSS-based schemes are being widely used on account of their low-power consumption and cost competitiveness because no extra devices are needed. However, these schemes suffer from poor localization accuracy due to additional signal attenuation resulting from transmission through walls and other big obstacles and severe RSS fluctuation due to multipath fading and indoor noise [14,15,16]. Moreover, the NLOS propagation property may seriously degrade the performance of AOA-based and TOA-based indoor localization due to the difficulty of detecting the direct signal path and measuring the time of flight of the signal, respectively [17]. Thus, the metric arrived at using the AOA, RSS or TOA solely is vulnerable to environmental changes [13]. To solve this problem, the authors of Reference [18] proposed to use the metric of RSS/TOA to conduct the localization based on the determining likelihood function, which is used to depict the relations between the measurements and distances. The authors of Reference [19] presented a scheme in which the metrics of RSS and AOA are integrated to restrain the NLOS fading. The authors of Reference [20] developed an efficient approach to localize the mobile sensors using the metrics of TOA and AOA with the help of multiple seeds adopted to obtain adequate observations [13]. Reference [21] proposed a RSS–AOA hybrid localization method to enhance the accuracy and robustness. Compared to the geometric positioning method based on single feature machines, this method utilized the slope (AOA) and the magnitude (RSS) between target and transmitter, thus the target coordinates could be determined. Nevertheless, the chance of error also increases with the increase in distance between anchor and target.

Some machine learning techniques are utilized in fingerprint localization. Well-known algorithms are the Nearest Neighbor (NN) [22], K Nearest Neighbor (KNN) [23], Weighted K Nearest Neighbor (WKNN) [24,25] and Support Vector Machines (SVM) [26] which belong to classification algorithms in machine learning. In the KNN algorithm [23], the prediction results can be obtained by selecting the nearest K samples in the training set according to the Euclidean distance between the position to be estimated and the known offline positions [27]. In the WKNN algorithm [24], the RSS collected by the nodes are compared to samples in the offline database, and the nodes’ positions are given by weighted combinations of the K nearest neighboring positions; the nearness indicator for this method is based on the Euclidean distance between RSSs [5]. In order to get better performance for the non-linear multi-class scenarios, SVM has been used in indoor wireless positioning in Reference [28]. It transforms the data into high-dimensional feature space by nonlinear transformation, and constructs the linear discriminant function in the high-dimensional space to realize the nonlinear discriminant function in the original space and has high computing complexity [29,30]. However, the results obtained by classification algorithm are discrete and struggle to meet the high precision requirements of indoor positioning. In order to improve the precision, some regression machine learning algorithms are applied. Support Vector Regression (SVR) [31,32,33] is used to find the positioning function that controls the accumulative error. However, the computational complexity of the SVR algorithm is cubic in the number of training data, because its solution process involves n-order positive definite matrix inversion [34]. Reference [35] proposed a deep-learning-based fingerprinting scheme which can fully explore the features of wireless channel data and obtain the optimal weights as fingerprints. However, the prediction performance of a deep neural network is highly dependent on the size of the training data set and may not be better than a machine learning algorithm when the number of training samples is small. Moreover, the training of a deep neural network requires a large amount of matrix operations and may be time-consuming and costly. The ridge regression algorithm [36] is another regression machine learning algorithm that has been used in localization [5]. Compared to the least squares method, the ridge regression algorithm is more reliable [37]. However, finding the optimal values for tuning regularization parameter and kernel parameter is the most complex part in terms of computations [5,37], and compared with WKNN and SVR, ridge regression has the best performance in terms of precision and robustness.

Increasingly researchers focus their attention on the Wi-Fi fingerprint localization [38] and Pedestrian Dead Reckoning (PDR) [39] localization system, which is based on inertial sensors for reasons of low cost, good compatibility, extendibility etc. However, the single-mode localization technology is unable to meet the demand of people in the complex indoor environment because of its own limitations. Thus, some fusion models, which are built based on two or more existing models, have been proposed. The most general fusion scheme is based on Wi-Fi fingerprint and PDR [40,41,42] since they possess complementary properties. For building the fusion model, Reference [43] makes use of the Kalman filter, which is the optimal filter for the linear model. However, since Kalman is a linear optimal filter, it cannot solve complex non-linear localization problems. To overcome this problem, the EKF (extended Kalman filter) [44] and UKF (unscented Kalman filter) [45,46], which are non-linear developments of the Kalman filter, are generally utilized. Compared with the Kalman filter, the particle filter [47] has better generalization ability, thus is more suitable for the non-linear problem. However, the localization model built with the particle filter is much more complicated, which could be destructive for real-time performance of the model. Besides this, some other methods, such as the Hidden Markov Model (HMM) and Conditional Random Field (CRF), are also utilized.

### 1.2. Motivation and Contribution

After summarizing the above references, we note that among the machine learning algorithms, regression methods are able to provide better performance in the accuracy of indoor localization. On the other hand, exploiting different features provides another way to improve the accuracy of localization. In order to address the above issue, we propose heterogeneous features fusion (HFF) machines to effectively improve localization. Multiple features fusion machine learning models are given. First, we propose the Heterogeneous Feature Fusion ridge regression (HFF-RR) model. The results show that precision is improved and the robustness to noise is also improved compared with Reference [5]. Second, in order to eliminate the bias caused by noisy data, we propose the heterogeneous feature selection (HFS) model by employing group LASSO [48]. Additionally, we have designed a fast algorithm to solve the model which combines the Newton iteration method with gradient descent. The algorithm is operated via the backtracking line search method, which accelerates convergence. Third, in order to separate the impact of each feature, we provide another machine model by using L1-Norm Penalty. Fourth, to reduce HFF-RR computational complexity, we simplify the HFF model as a set of underdetermined equations, then transform it as a constraint optimization problem. Numerical results show that, compared with other proposed learning methods, ours has the lowest computation complexity but relatively high accuracy of localization. We compare our proposed localization algorithms with two state-of-the-art localization algorithms based on feedback and correction of Wi-Fi signals and PDR information fusion: EKF and UKF [43]. Simulation data shows that our HFF model outperformances EKF and UKF when considering both localization accuracy and time efficiency.

### 1.3. Organizations

The rest of the paper is organized as follows. The general model for localization is firstly introduced in Section 2. Our proposed hybrid features machine learning models and algorithm are presented and compared in Section 3. In Section 4, the real data collection procedure is elaborated, and then the performance of the proposed methods is evaluated on both simulated and real-world signals. Finally, Section 5 concludes the work.

## 2. Machine Learning for Indoor Localization

**Figure 1:**
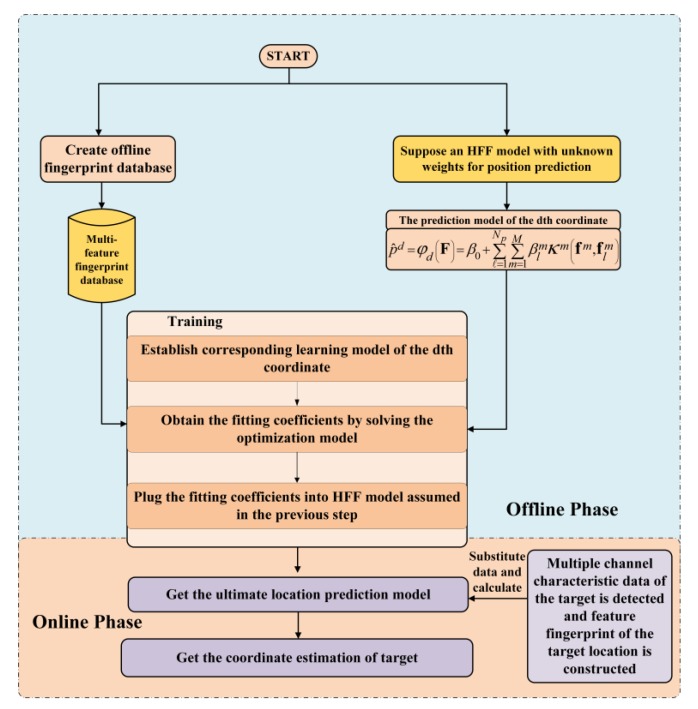
Proposed method for predicting target location in the offline and online phase.

We consider an environment of D dimensions in which the points denoted by $p=[p^{1},\ldots ,p^{D}]$ are filled. In addition, two types of sensors are considered: Access Point (AP) and mobile nodes. APs as signal emission nodes are evenly distributed in the D-dimensional space, denoted by $a_{r}=[a_{r}^{1},\ldots ,a_{r}^{D}]$, $r\in \left\{1,\ldots ,N_{a}\right\}$, where $N_{a}$ is the total number of APs. Mobile nodes are used for receiving signals, whose locations are known as training samples, denoted by $p_{l}=[p_{l}^{1},\ldots ,p_{l}^{D}]$,$l\in \left\{1,\ldots ,N_{p}\right\}$, where $N_{p}$ is the total number of mobile nodes. The fingerprinting localization scheme consists of two phases, namely offline and online phases, respectively, as illustrated in Figure 1. In the offline phase, the broadcast signals are transmitted by APs at a constant initial power. Meanwhile, each sensor placed at a known position is used to detect the signal features transmitted by all $N_{a}$ APs. Let $F_{l}=[f_{l}^{1},\ldots ,f_{l}^{M}]$ be the feature matrix at the offline training position $p_{l}$, where $f_{l}^{m}={\left[f_{l1}^{m},\ldots ,f_{lN_{a}}^{m}\right]}^{T}$ is a $N_{a}$ column vector denoting the *m*th feature of the lth training sample. m is the index of feature, m∈{1,…,M}. Hence, the offline fingerprint database includes M features which can be expressed as ${\left\{(F_{l};p_{l})\right\}}_{l=1}^{N_{p}}$.

Therefore, an offline training set ${\left\{(F_{l};p_{l}^{d})\right\}}_{l=1}^{N_{p}}$ is available to learn the model for the estimation of the *d*th dimensional coordinate $p^{d}$, where d∈{1,…,D}. The function used to estimate $p^{d}$ would be indicated as follows

(1) $$\varphi_{d}\left(\cdot \right):{\mathbb{R}}^{N_{a}\times M}\mapsto \mathbb{R},d\in \left\{1,\ldots ,D\right\}\;$$

Therefore, the estimated *d*th coordinate can be obtained by

(2) $${\hat{p}}^{d}=\varphi_{d}\left(F\right)\;$$

As one of the most popular machine learning tools, kernel machine is properly effective for learning a nonlinear function [49]. In kernel machine, the input data is implicitly embedded into a high-dimensional space by a nonlinear mapping. Linear functions in the transformed kernel space are naturally equivalent to a rich class of nonlinear functions in the original data space, which constitute the so-called reproducing kernel Hilbert space (RKHS) [50,51]. We make $\varphi_{d}(\cdot )$ to be a kernel-based machine learning model, and the reproducing kernel function could be defined as

$$K:{\mathbb{R}}^{N_{p}}\times {\mathbb{R}}^{N_{p}}\mapsto \mathbb{R}\;$$

In practice, the classic kernels functions are such as the linear, polynomial and Gaussian kernel functions. Here, we select the Gaussian kernel functions

(3) $$K^{m}(f^{m},f_{l}^{m})=\exp\left(-\frac{1}{2\sigma_{m}^{2}}{\left\|f^{m}-f_{l}^{m}\right\|}_{2}^{2}\right)\;$$

where $f^{m}$ is a $N_{a}$ column vector denoted the *m*th feature of input sample. Additionally, for each feature m, the similarity metric between two samples is represented by $K^{m}(f^{m},f_{l}^{m})$. Then we model the regression function by

(4) $${\hat{p}}^{d}=\varphi_{d}(F)=\beta_{0}+\sum_{l=1}^{N_{p}}\sum_{m=1}^{M}\beta_{l}^{m}K^{m}(f^{m},f_{l}^{m})\;$$

where $\beta_{l}^{m}$ is the unknown kernel regression coefficient associated with the *l*th sample and the *m*th feature. $\beta_{0}$ is the bias. The new model provides a flexible way to fuse multiple features, where the fusion weights are formulated as part of the kernel regression coefficients and will be adaptively estimated from the data.

To solve $\varphi_{d}(F)$, we minimize the following loss function

(5) $$C^{d}=L((p_{1}^{d},\varphi_{d}(F_{1})),\ldots ,(p_{N_{p}}^{d},\varphi_{d}(F_{N_{p}})))+\lambda R(\varphi_{d})\;$$

where L denotes the empirical loss over training set, λ is tuning parameter, and the regularization term R is usually a monotone function of the RKHS norm of $\varphi_{d}$. The regression loss is chosen because: (a) it produces a relatively good performance on localization accuracy while the other machine learning algorithms cannot compare; (b) it gives continuous results which are much more accurate than classification results; (c) ridge regression loss function is sometimes differentiable, which is preferred for optimization. We will show later that such a loss function gives rise to an efficient learning algorithm.

## 3. Fusion Machines Models and Algorithms

In this section, we need to find a set of functions $\varphi_{d}(F),d\in \{1,\ldots ,D\}$, which associates each feature matrix F to the corresponding coordinates $p^{d}$. We propose several learning models and efficient algorithms to find the appropriate $\varphi_{d}(F)$.

### 3.1. Heterogeneous Feature Fusion Ridge Regression (HFF-RR)

In this subsection, we define the function $\varphi_{d}(\cdot )$ to minimize the following regularized risk:

(6) $$\underset{\varphi_{d}}{\min}\left\{\sum_{j=1}^{N_{p}}{\left(p_{j}^{d}-\varphi_{d}(F_{j})\right)}^{2}+\lambda \sum_{l=1}^{N_{p}}{\left\|\beta_{l}\right\|}_{2}^{2}\right\}\;$$

where $\beta_{l}$ are the corresponding coefficients of the lth sample, denoted by.$\beta_{l}=[\beta_{l}^{1},\ldots ,\beta_{l}^{M}]$

Let β be a $N_{p}\times M+1$ column vector which consists of scalar $\beta_{0}$ and $N_{p}$ column vectors, denoted by $\beta ={\left[\beta_{0},\beta_{1}^{T},\ldots ,\beta_{N_{p}}^{T}\right]}^{T}$. By plugging (4) into (6), the optimization problem (6) could be written in the following matrix format:

(7) $$\underset{\beta }{\min}\{{\left(p^{d}-K\beta \right)}^{T}(p^{d}-K\beta )+\lambda \beta^{T}E^{\prime }\beta \}\;$$

where Κ is denoted by $Κ=\left[\begin{array}{cc} l & {Κ}^{\prime } \end{array}\right]$, l is the $N_{p}-\mathrm{by}-N_{p}\times M+1$ identity matrix, and ${Κ}^{\prime }$ is a $N_{p}-\mathrm{by}-N_{p}\times M$ matrix whose $\left(l,l^{\prime }\times m\right)$ entry is ${Κ}^{m}(f^{m},f_{l}^{m})$, $E^{\prime }$ can be denoted by $E^{\prime }=\left[\begin{array}{cc} 0 & 0^{T}\\ 0 & I \end{array}\right]$.

The solution is obtained by taking the derivative with respect to β and setting it to zero:

(8) $${Κ}^{T}(Κ\beta -p^{d})+\lambda E^{\prime }\beta =0\;$$

This leads to the following form of β:

(9) $$\beta ={\left(K^{T}K+\lambda E\right)}^{-1}K^{T}p^{d}\;$$

It is noticed that we can find an appropriate regularization coefficient λ to make the matrix $\left(K^{T}Κ+\lambda E^{\prime }\right)$ to be nonsingular.

### 3.2. Heterogeneous Feature Selection using Group LASSO Penalty (HFS-GLP)

Due to the large number of parameters, in order to prevent an overfitting problem and try to remove the noisy samples, we employ the group LASSO [52] regularization. To learn a group sparse model, the final cost function is defined as

(10) $$\underset{\beta }{\min}\left\{\sum_{j=1}^{N_{p}}{\left(p_{j}^{d}-\varphi_{d}(F_{j})\right)}^{2}+\lambda \sum_{l=1}^{N_{p}}{\left\|\beta_{l}\right\|}_{2}\right\}\;$$

where ${\left\|\cdot \right\|}_{2}$ denotes the $l_{2}$ norm. The group LASSO leads to a sparse constraint at group level by combining $l_{1}$ norm and $l_{2}$ norm, and it uses $l_{2}$ norm within a group and $l_{1}$ norm between groups.

#### An Efficient Iterative Optimization (EIO) Algorithm

Since the group LASSO regularization term is not differentiable, an iterative algorithm should be employed to minimize the model. In this paper, we proposed an efficient approach to solve our optimization problem (10). A gradient descent method the step size of which is acquired by the backtracking line search is a desirable algorithm. However, due to its extremely slow convergence near the point at which the target function achieves its minimum value, it is still hard to obtain the optimal solution. Meanwhile, the Newton iterative algorithm has quadratic convergence speed. The algorithm could reach convergence by only one iteration for the optimization problem when its Hessian matrix is positive definite. Therefore, we propose an improved learning algorithm which combines these two iterative algorithms. We call it Efficient Iterative Optimization (EIO). For simplicity, we outline the framework of EIO in Algorithm 1

EIO consists of two phases. In the first phase, we set an arbitrary point as the initial point and then obtain a point quite close to the optimal solution by using the gradient descent method the step size of which is acquired by the backtracking line search, executed by repeating

(11) $$\beta^{\left(k+1\right)}=\beta^{\left(k\right)}-\alpha_{k}\nabla C^{d}(\beta^{\left(k\right)})\;$$

where k denotes the kth iteration. The step size is obtained by using the backtracking line search method. In this method, $\alpha_{k}$ is updated by $\alpha_{k}=\nu \alpha_{k}$,ν∈(0,1) until the Armijo rule is met, i.e., the following inequality holds:

(12) $$C^{d}(\beta^{\left(k\right)}-\alpha_{k}\nabla C^{d}(\beta^{\left(k\right)}))\leq C^{d}(\beta^{\left(k\right)})-\mu \alpha_{k}{\left\|\nabla C^{d}(\beta^{\left(k\right)})\right\|}_{2}^{2}\;$$

where μ is an arithmetic number and μ∈(0,0.5).

In the second phase, we use the Newton iterative method. We let the result of the previous phase be the initial point. In practice, the Newton iterative method exploits first-order and second-order information of the cost function to get the optimal β. Within the kth iteration, it is calculated by

(13) $$\beta^{\left(k\right)}=\beta^{\left(k-1\right)}-H_{k-1}^{-1}\nabla C^{d}(\beta^{\left(k-1\right)})\;$$

where $H_{k-1}$ is the Hessian matrix of the target function $C^{d}$ at the point $\beta^{\left(k-1\right)}$.

**Algorithm 1**: Outline of EIO algorithm1: let $\beta^{\left(0\right)}$ be the initial point 2: let $t_{M}$ be the number of iterations, do the gradient descent method whose step size is acquired by the backtracking line search, and output ${\beta^{\prime }}_{\mathrm{opt}}$. 3: let ${\beta^{\prime }}_{\mathrm{opt}}$ be the initial point, do Newton iterative algorithm until the algorithm is converged. **Output**: Obtain the precision $\beta_{\mathrm{opt}}$

**Figure 2:**
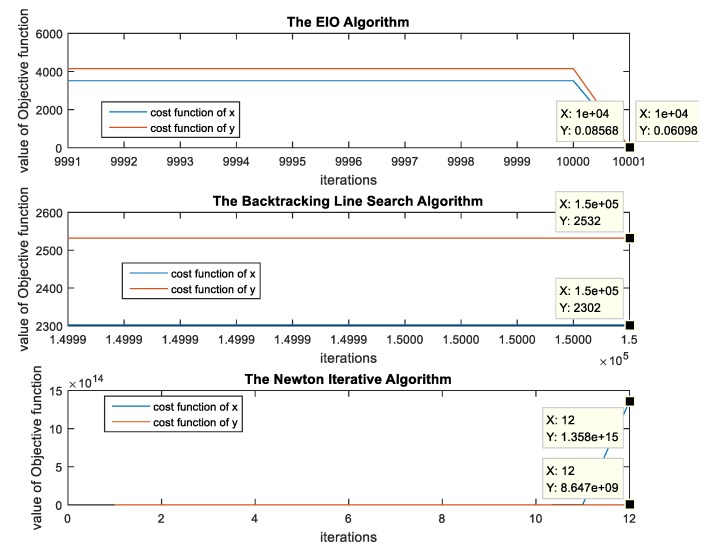
The comparison among the optimized objective function curves of the EIO algorithm, the individual Newton method, and the backtracking line search gradient decent method.

We illustrate the effectiveness of the EIO algorithm through simulation experiments. In this simulation, the three iterative algorithms use the same initial value points. The simulation results are shown in Figure 2, which demonstrates the comparison among the objective function curves of these three iterative algorithms, i.e., the EIO algorithm and the individual Newton method and backtracking line search gradient decent method. The simulation results show that the objective function reaches 0.08 after 10,001 iterations in the EIO algorithm. However, the backtracking line search gradient descent algorithm still does not converge after more than 100,000 iterations, and the Newton iteration method does not converge.

### 3.3. Heterogeneous Feature Selection Using L1-Norm Penalty (HFS-LNP)

The model in Equation (10) removes the noisy samples which contain multiple features. In this subsection, we propose a learning model to remove the ruined features instead of multiple features of the sample. The corresponding optimization problem could be expressed as follow:

(14) $$\underset{\beta }{\min}\left\{\frac{1}{2}{\left\|p^{d}-K\beta \right\|}_{2}^{2}+\lambda {\left\|\beta \right\|}_{1}\right\}\;$$

As the interpretation of Reference [53], SALSA (Split Augmented Lagrangian Shrinkage Algorithm) which combines the augmented Lagrangian approach and the variable splitting technique is available for solving linear inverse problems with sparse regularization. By applying variable splitting to Equation (14), the constraint optimization problem is written as:

(15) $$\begin{array}{l} \underset{x,u}{\min}\left\{\frac{1}{2}{\left\|p^{d}-K\beta \right\|}_{2}^{2}+\lambda {\left\|u\right\|}_{1}\right\}\\ \begin{array}{cccc} s.t. & & & u-\beta =0 \end{array} \end{array}$$

We exploit the conclusion of Reference [53], and use the following solution to solve the optimization problem in Equation (15)

initialized   μ>0,drepeat(16a)v←soft(β+d,λl/μ)−d(16b)β←(KHK+μI)−1(KHpd+μv) (16c)d←d−u+β end

where v=u−d and the operator $K^{H}$ is the Hermitian conjugate transformation of K.

### 3.4. Heterogeneous Feature Fusion by Solving Underdetermined Equations (HFF-UE)

For the algorithm proposed in previous sections, finding the optimal values for tuning regularization parameter and kernel parameter is the most complex part in terms of computations. Its computational cost would be increased by ten times as one parameter is added. In this subsection, we try to remove the regularization parameter to reduce the computation complexity. We formulate the training set ${\left\{(F_{l};p_{l}^{d})\right\}}_{l=1}^{N_{p}}$ as a group of equations

(17) $$\{\begin{array}{c} \varphi_{d}\left(F_{1}\right)=p_{1}^{d}\\ \vdots \\ \varphi_{d}\left(F_{l}\right)=p_{l}^{d}\\ \vdots \\ \varphi_{d}\left(F_{N_{p}}\right)=p_{N_{p}}^{d} \end{array}\;$$

Since the number of features in Equation (17) are much more than the number of equations, it is underdetermined. It could be rewritten as

(18) $$K\beta =p^{d}\;$$

where $p^{d}$ is a $N_{p}$ column vector which is denoted by $p^{d}={\left[p_{1}^{d},\ldots ,p_{N_{p}}^{d}\right]}^{T}$ and Κ is a “wide” matrix whose columns more than the rows, its rows are linearly independent. In this case, we formulate it as an optimization problem.

(19) $$\begin{array}{cc} & \underset{\beta }{\min}{\left\|\beta \right\|}_{2}^{2}\\ s.t. & p^{d}=K\beta \end{array}\;$$

By using Lagrange multipliers, we can derive the closed-form solution in Equation (20).

(20) $$\beta =K^{T}{\left(KK^{T}\right)}^{-1}p^{d}\;$$

### 3.5. The Relationship between the Proposed Four Learning Models

In this subsection, we address the differences and connections between our models. Table 1 lists the formulas for the four models and shows the performance of the four models in terms of precision, time efficiency and sparsity. These four learning models consist of the fitness term L and the penalty term R. The fitness terms are based on the smallest square error criterion. The penalty terms are diverse due to the different goals in positioning performance. HFF-RR applied L2-norm penalty term. Since the cost function is differentiable, there is an exact solution of this model which leads to the highest accuracy and robustness in positioning. In order to remove the noisy samples, the group LASSO which combines L1-norm and L2-norm is selected as the penalty term in HFS-GLP. HFS-LNP removes the ruined features by using L1-norm penalty term. Since L1-norm term is not differentiable everywhere, the optimal solution would be obtained by the iterative algorithm. Considering the computational burden, we propose an efficient approach to solve our optimization problem. Since the solution is calculated by iterations, the performance in terms of precision and robustness are poor in positioning. Furthermore, iterative computation leads to a reduction in computational efficiency. In HFF-UE, the optimization problem model is transformed from the Underdetermined Equations. It removes the regularization parameter so that the computational complexity is significantly reduced in the cross-validation phase. Therefore, it has the highest computation efficiency.

## 4. Numerical Analysis and Results

In this section, we evaluate the performance of the proposed approaches by simulation with real data. The system is reviewed first with the data collection and zone division, then computational efficiency and accuracy of the approaches are evaluated by comparison among different machine learning based popular methods and other popular data fusion methods. In the second part, the proposed heterogeneous feature machine is compared with the RSS-based kernel machines in different noise scenarios.

### 4.1. Real Experiment Setup

**Figure 3:**
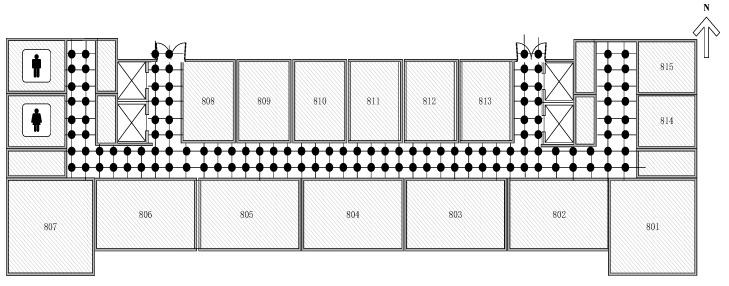
Floor plan of experiment area and anchor distribution.

To test the performance of the proposed models and algorithms, we did experiment in a school building. The floor plan is shown in Figure 3. The experiment area includes a long west-east oriented aisle and four shorter north-south oriented aisles. The long aisle is around 40 m while the shorter aisle is nearly 8.5 m. There are over ten APs arranged in the area with uniform specifications but unknown position. The direction from east to west is marked as X axis. The direction from south to north is marked as Y axis. The anchor points are set symmetrically with a 1.2 m spacing. There are 126 anchor points in total. In the office stage, we use TL-WN823N USB wireless network adapter which is compatible with the IEEE 802.11 n/g/b standard. The frequency of the system is operated on 2.4 GHz. In order to have enough data to do the simulation, we scan Wi-Fi RSS information at every anchor point 100 times, at a sampling interval of 1 s. The collected data is stored in text files. We import these data and perform simulation experiments on version 2015a of Matlab on a Sony laptop with Windows 7 and Intel^®^ Core™ i5 CPU.

### 4.2. Localization Accuracy and Computational Cost Evaluation

**Figure 4:**
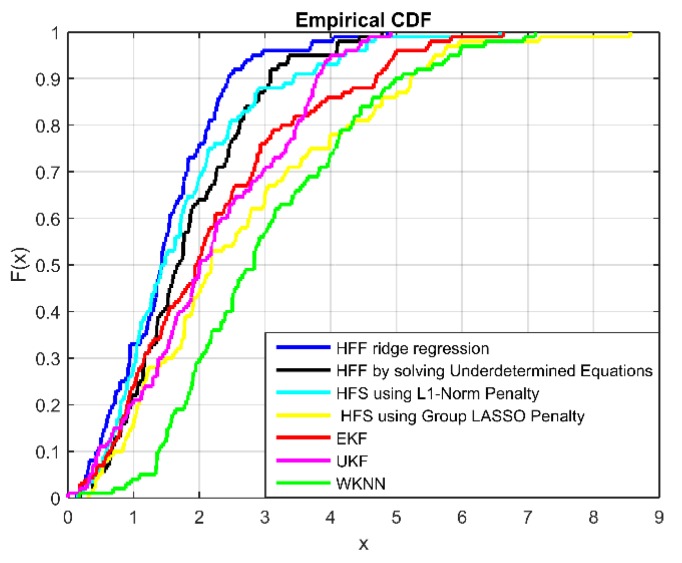
Comparison of Localization results in term of CDF.

**Figure 5:**
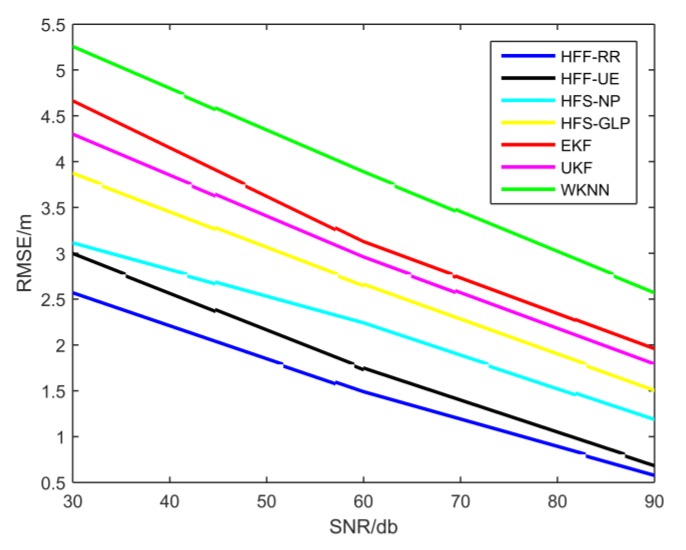
Root Mean Square Error (RMSE) as Signal Noise Ratio (SNR).

In this subsection, we mainly discuss the performance of the proposed four fusion machine models in terms of positioning accuracy and time efficiency. Meanwhile, we compare them with two state-of-the-art localization algorithms based on feedback and correction of Wi-Fi signals and PDR information fusion: EKF and UKF [43]. To evaluate the performance of various localization algorithms in terms of precision and time efficiency, the corresponding simulation results are recorded in Figure 4 and Figure 5 and Table 2. Figure 4 demonstrates the cumulative distribution function (CDF) of position estimation error of each positioning algorithm. The Figure 5 shows the root mean square error (RMSE) in terms of Signal Noise Ratio (SNR). The RMSE, running time of parameters optimization and corresponding optimal parameter are shown in Table 2. In terms of positioning accuracy, EKF and UKF have higher localization accuracy and localization stability than RSS-based Wi-Fi fingerprint location algorithm i.e., WKNN. Table 2 shows that the average error of EKF and UKF is about 3 m, while WKNN is about to reach 4 m. In Figure 4, the positioning error probability of EKF and UKF is significantly lower than WKNN. Although EKF and UKF improve positioning accuracy and stability, our proposed localization algorithms outperform them in terms of positioning accuracy and positioning stability. From Table 2, the positioning accuracy of the four positioning algorithms proposed in this paper is higher than EKF and UKF. From Figure 4, the probability of HFF-RR positioning error at 3 m reaches 95%, HFF-UE and HFS-LNP are close to 90%, while EKF and UKF are less than 80%. From the results in Figure 5, we can observe that the value of RMSE decreases as the SNR increases. In terms of time efficiency, since finding the optimal value of the tuning parameters is the most complex part in terms of computations, we measured the average running time for the parameters optimization phase for each algorithm. To reduce the computational complexity of EKF and UKF, we used the WKNN clustering algorithm to cluster the Wi-Fi fingerprint database. The K parameter denotes the number of classifications in WKNN. From Table 2, we observe that EKF and UKF are both faster than other algorithms, and the HFF-UE algorithm performs slightly slower than EKF and UKF in terms of time efficiency. However, the HFF-UE algorithm outperforms EKF and UKF in terms of positioning accuracy. Compared to the HFF-RR algorithm with the highest accuracy, the accuracy of HFF-UE algorithm is only slightly poorer, but the time complexity is much lower than that of HFF-RR. Therefore, the HFF-UE algorithm is a good localization model while considering both localization accuracy and time efficiency. Another point worth noting from Table 2 is that the performance of the first two learning algorithms outperform the follow two algorithms in terms of whether precision or computation complexity. The large number of iterations in the follow two algorithms contribute to large amounts of running time in parameters optimization phase. Moreover, the result of an iteration is an approximation instead of an exact value of the optimal value. 

### 4.3. Heterogeneous Feature Fusion Machines vs. Single feature machines

In this subsection, we compare our proposed heterogeneous feature machine with the RSS-based single feature machine proposed in Reference [5] in different noise scenarios. Same as the previous subsection, simulations are run on version 2015a of Matlab on a SONY laptop with Windows 7 and Intel^®^ Core™ i5 CPU. The simulation is set up in a 100 m×100 m 2D environment where 16 static APs and 100 offline training positions are set. The offline training set is denoted as ${\left\{(F_{l};p_{l})\right\}}_{l=1}^{100}$ where matrix $F_{l}=[f_{l}^{1},f_{l}^{2}]$ describes the features of the lth known training sample whose position is denoted as $p_{l}=[p_{l}^{1},p_{l}^{2}]$. As the component of $F_{l}$, $f_{l}^{m}={\left[f_{l1}^{m},\ldots f_{l16}^{m}\right]}^{T}$ represents the features which are received at location $p_{l}$. We utilize RSS and TOA features. It represents RSS when m=1 or represents TOA feature when m=2.

The entries of $f_{l}^{1}$ are the RSS values at position $p_{l}$ emitted by different APs. They are generated utilizing the well-known Okumura–Hata model [54]. The power $f_{lr}^{1}$ received at position $p_{l}$ from the AP $a_{r}$ can be expressed by:

(21) $$f_{lr}^{1}=\rho_{0}-10n_{p}\log_{10}\left\|a_{r}-p_{l}\right\|+\varepsilon_{lr}\;$$

where $\rho_{0}$ is the initial power set to a fixed value 150 dBm, $n_{p}$ is the path-loss exponent set to 4, $\left\|a_{r}-p_{l}\right\|$ is the Euclidian distance between the position $p_{l}$ and the position $a_{r}$, and $\varepsilon_{lr}$ is the noisy in indoor wireless channel.

For the element of $f_{l}^{2}$, $f_{lr}^{2}$ is the propagation time of the signal transmission from the position $a_{r}$ to the position $p_{l}$. Its value can be obtained by the following formula:

(22) $$f_{lr}^{2}=\left\|a_{r}-p_{l}\right\|/c+\tau_{lr}\;$$

where c is $3.0\times 10^{8}m/s$ which is the velocity of light, $\tau_{lr}$ is the time delay caused by the propagation of light.

For any learning algorithms, it is necessary to choose the optimum parameter for accurate positioning. We use the cross-validation to choose the optimal parameters. We use k-fold cross-validation, the basic form of cross-validation, consists of separating the data into k probably equally sized folds. At each iteration, k–1 folds are used for training and the rest for validation. For each group of parameters which just provides for a certain learning algorithm, the performance are measured by the mean error of validation set in k iterations. The optimal tuning parameters are these may contribute to the minimum mean error of validation. The value of k is set to 10.

For each learning model, our simulation experiment can be divided into the following steps:

- Optimizing the relevant parameters using 10-fold cross-validation method.
- Learning the location model $\psi \left(\cdot \right)=\left[\varphi_{1}\left(\cdot \right),\varphi_{2}\left(\cdot \right)\right]$ in training set by using current learning algorithm.
- Validating the model learned from the previous step in validation set.

**Figure 6:**
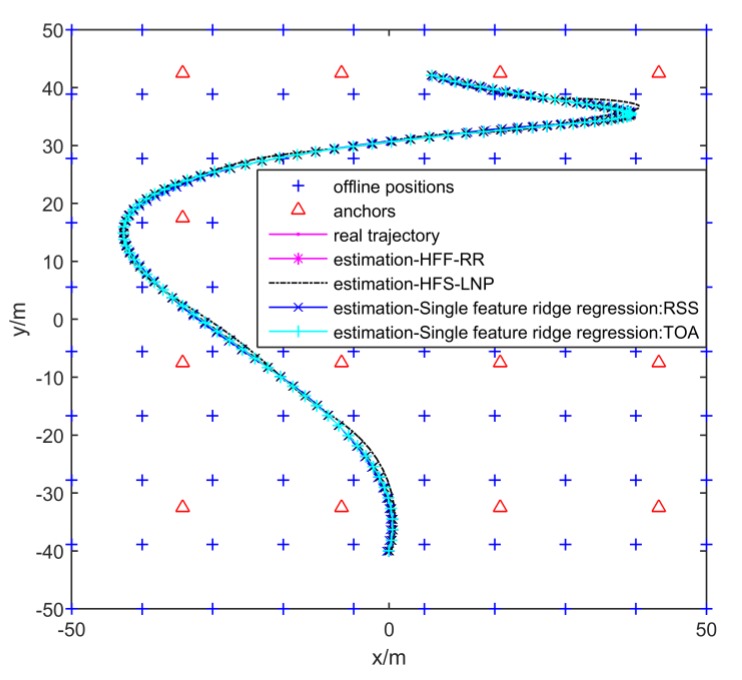
Estimation of trajectory simulated several times by heterogeneous feature machine and single feature-based kernel machines learned by different learning algorithms in absence of noise.

Figure 6 demonstrates the comparison between the estimated curves of the generated trajectory in several simulations by the heterogeneous feature machine and single feature-based kernel machine mentioned in Reference [5]. The heterogeneous feature machine learned via HFF-RR and HFS-LNP in the absence of noise. The single feature-based kernel machine learned by using single feature ridge regression. The estimation error, measured by the root mean squared distance between the exact positions and the estimated ones, as well as each optimal parameter, are shown in Table 3. We notice that the heterogeneous feature machine outperforms single feature-based kernel machine whether with HFF-RR or HFS-LNP.

To prove the robustness of the proposed model, we carried out simulations in certain noisy scenarios. We set RSS with Gaussian random noise. We set TOA with non-line-of-sight errors. The estimation error and optimal parameters of our proposed model HFF-RR and the model introduced by Reference [5] are shown in Table 4. We consider three kinds of noise scenarios: noisy RSS but true TOA; noisy TOA but true RSS; and finally noisy TOA and noisy RSS. The results indicate that the mean error of localization using heterogeneous feature machine is far less than the single feature-based kernel machine in noise conditions. 

It is noted that the HFF model (4) adaptively selects features based on noise conditions in Table 3 and Table 4. From Table 3 and Table 4, we observed that $\sigma_{m}$ corresponding to the mth feature increase as the noise increases. According to Reference [3], increasing the value of $\sigma_{m}$ will reduce the correlation between the input vector $f^{m}$ and the value of kernel function. Therefore, the impact of the mth feature on the position coordinate estimation will decrease.

## 5. Conclusions

In this paper, we proposed several heterogeneous feature machine learning models for localization, namely, HFF-RR, HFS-GLP, HFS-LNP and HFF-UE. In the model of HFS-GLP, in order to solve the corresponding optimization problem, we proposed a novel iterative algorithm which combines the Newton iteration method and gradient descent. From the aspect of time efficiency, HFF-UE model shows the best performance among all four models. From the aspect of localization accuracy, HFF-RR model provides the highest precision and robustness to noise. From the aspect of removing outlier noise, HFS-GLP model can remove the noise at the sample level and the HFS-LNP can remove the noise at the feature level. In contrast to other latest data fusion method for indoor localization, the proposed methods outperform others in computational cost and localization accuracy by doing real experiments. On the other hand, in contrast to the single feature-based kernel machine, in our proposed localization model based on heterogeneous features, the accuracy is improved significantly. In the case of relatively poor channel environment, the positioning accuracy of the proposed model can still be maintained at a high level.

## Figures and Tables

**Table 1:** The relationship between the proposed four learning model.

Learning Model	Error (m)	Time (ms)	Sparsity
$\underset{\varphi_{d}}{\min}\left\{\sum_{j=1}^{N_{p}}{\left(p_{j}^{d}-\varphi_{d}(F_{j})\right)}^{2}+\lambda \sum_{l=1}^{N_{p}}{\left\|\beta_{l}\right\|}_{2}^{2}\right\}$	0.57 ± 0.55	1.0 × 10^5^ $\pm \;1.5\times 10^{4}$	No sparsity
$\underset{\beta }{\min}\left\{\sum_{j=1}^{N_{p}}{\left(p_{j}^{d}-\varphi_{d}(F_{j})\right)}^{2}+\lambda \sum_{l=1}^{N_{p}}{\left\|\beta_{l}\right\|}_{2}\right\}$	1.43 ± 1.23	4.27 $\times 10^{8}$ ± 2.0 $\times 10^{7}$	Sample-level sparsity which can denoise at the sample level
$\underset{\beta }{\min}\left\{\frac{1}{2}{\left\|p^{d}-K\beta \right\|}_{2}^{2}+\lambda {\left\|\beta \right\|}_{1}\right\}$	1.36 ± 0.88	5.21 $\times \;10^{7}$ $\pm \;2.50\times 10^{6}$	Feature-level sparsity which can denoise at the feature level
$\begin{array}{cc} & \underset{\beta }{\min}{\left\|\beta \right\|}_{2}^{2}\\ s.t. & p^{d}=K\beta \end{array}$	1.04 ± 0.71	5.01 $\times \;10^{3}$ $\pm 1.50\times 10^{3}$	No sparsity

**Table 2:** Estimation error and computation complexity for different algorithms under TOA & RSS noise conditions.

Learning Model and Algorithm	$\lambda N_{p}$	$\sigma_{1}$	$\sigma_{2}$	K	Average Running Time of Parameters Optimization (s)	Error (m)
HFF-RR	2^−5^	2^6^	2^9^	—	102.59	1.5238
HFF-UE	—	2^6^	2^9^	—	4.35	1.8756
HFS-LNP	2^4^	2^6^	2^9^	—	56,635.99	2.2409
HFS-GLP	2^−24^	2^20^	2^10^	—	407,395.65	2.6627
EKF	—	—	—	5	1.35	3.1274
UKF	—	—	—	5	1.78	2.9601
WKNN	—	—	—	5	0.99	3.8901

**Table 3:** Performance comparison of heterogeneous feature machine and single feature-based kernel machine learned by different algorithms in absence of noise.

Learning Model and Algorithm	Prediction Model	$\lambda N_{p}$	$\sigma_{1}$	$\sigma_{2}$	Error (m)
Single feature ridge regression	RSS-based kernel machine	2^−32^	2^7^	—	0.1995
	TOA-based kernel machine	2^−35^	—	2^10^	0.0258
HFF-RR	heterogeneous feature machine	2^−34^	2^13^	2^10^	0.0215
HFS-LNP	heterogeneous feature machine	2^3^	2^6^	2^10^	0.6698

**Table 4:** Performance comparison of heterogeneous feature machine and single feature-based kernel machine learned by HFF-RR algorithms under noise conditions.

Noise Conditions	Prediction Model	$\lambda N_{p}$	$\sigma_{1}$	$\sigma_{2}$	Error (m)
Noisy RSS and True TOA	RSS-based kernel machine	2^−5^	2^6^	—	1.8756
	heterogeneous feature machine	2^−34^	2^23^	2^10^	0.0137
Noisy TOA and True RSS	TOA-based kernel machine	2^−7^	—	2^9^	2.0300
	heterogeneous feature machine	2^−22^	2^6^	2^33^	0.2288
Noisy TOA and Noisy RSS	heterogeneous feature machine	2^−5^	2^6^	2^9^	1.2569
